# Supplementary material for: Evolutionary origin and structural ligand mimicry by the inserted domain of alpha-integrin proteins
Source: bioRxiv. 2023 Nov 6:2023.11.05.565221. Preprint. [Version 1] doi: 10.1101/2023.11.05.565221 (PMC10659397; doi:10.1101/2023.11.05.565221)
Supplement: 1 [file NIHPP2023.11.05.565221V1-supplement-1.pdf]

bioRxiv preprint doi: https://doi.org/10.1101/2023.11.05.565221; this version posted November 6, 2023. The copyright holder for this preprint (which was not certified by peer review) is the author/funder, who has granted bioRxiv a license to display the preprint in perpetuity. It is made available under aCC-BY 4.0 International license.

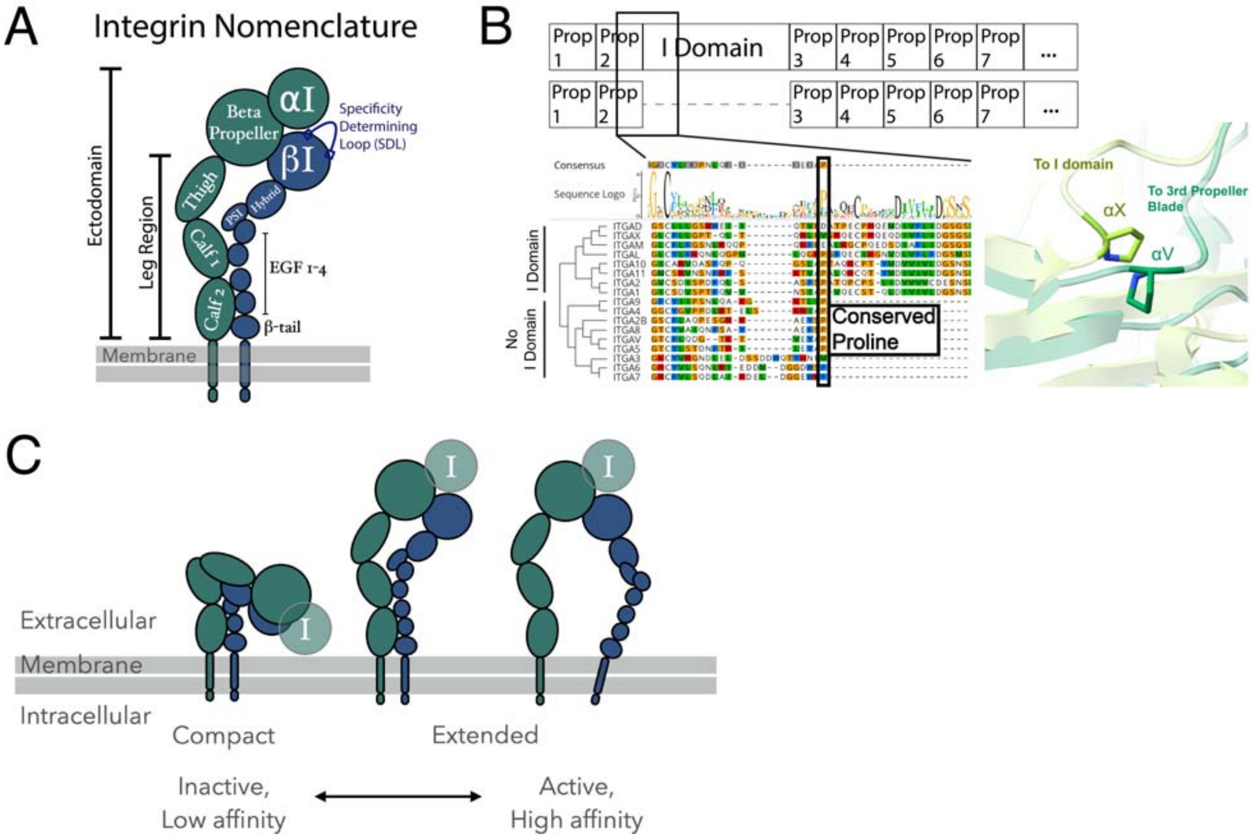

Supplemental Figure 1. Structural features of integrin molecules. A, A schematic of integrin ectodomain domain and the beta-propeller organization. The I domain has inserted between the second and third of seven β-propeller domain repeats in some α-integrin genes. **B,** An alignment of all human integrin α subunits (except αE) shows insertion of the I domain in the ancestral integrin gene occurred immediately following a conserved proline at the end of the second β-propeller blade. Slightly offset structural overlays of the I domain-less integrin αV (dark green, PDB 1L5G[52]) and I domain-containing αX (lime green, PDB 4NEH[11]) with their respective conserved prolines displayed. **C,** Integrin conformations range from a compact, inactive state with low ligand affinity (left) to an extended-open, active state with high ligand affinity (right). Approximate hypothesized I domain locations are shown as semi-transparent.

bioRxiv preprint doi: https://doi.org/10.1101/2023.11.05.565221; this version posted November 6, 2023. The copyright holder for this preprint (which was not certified by peer review) is the author/funder, who has granted bioRxiv a license to display the preprint in perpetuity. It is made available under aCC-BY 4.0 International license.

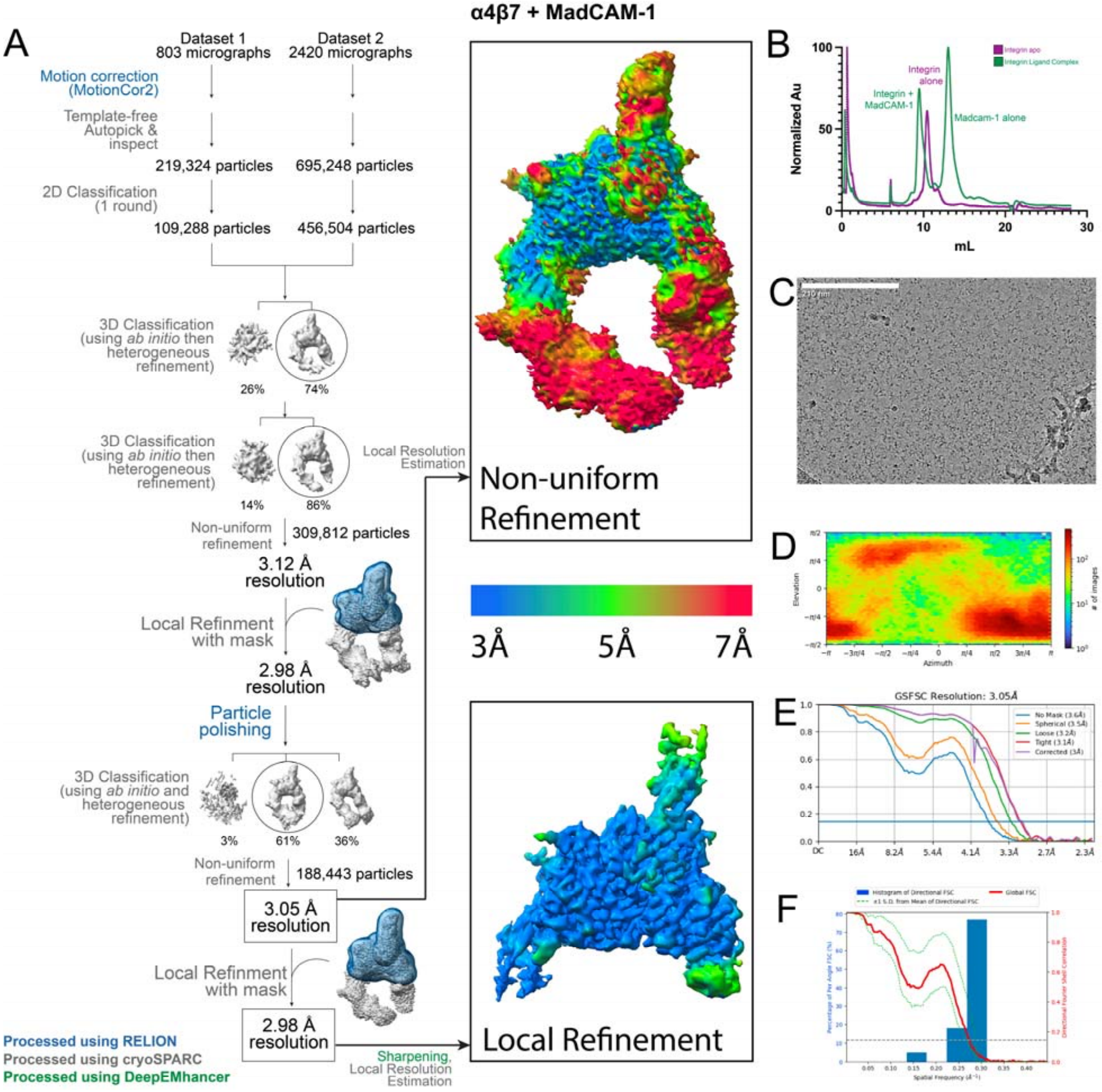

Supplemental Figure 2. Data processing schematic for the α4β7:MadCAM-1 complex. A, A flowchart for the data processing pipeline of α4β7:MadCAM-1. Both the global and local refinements were used for model building. **B,** Size exclusion chromatography traces showing peak shift for ligand-bound integrin. **C,** Representative micrograph with 210nm scale bar, **D,** orientational distribution plot, **E,** gold-standard Fourier Shell Correlation (GSFSC) plot, and **F,** three-dimensional Fourier Shell Correlation (3DFSC) plot for the globally refined map.

bioRxiv preprint doi: https://doi.org/10.1101/2023.11.05.565221; this version posted November 6, 2023. The copyright holder for this preprint (which was not certified by peer review) is the author/funder, who has granted bioRxiv a license to display the preprint in perpetuity. It is made available under aCC-BY 4.0 International license.

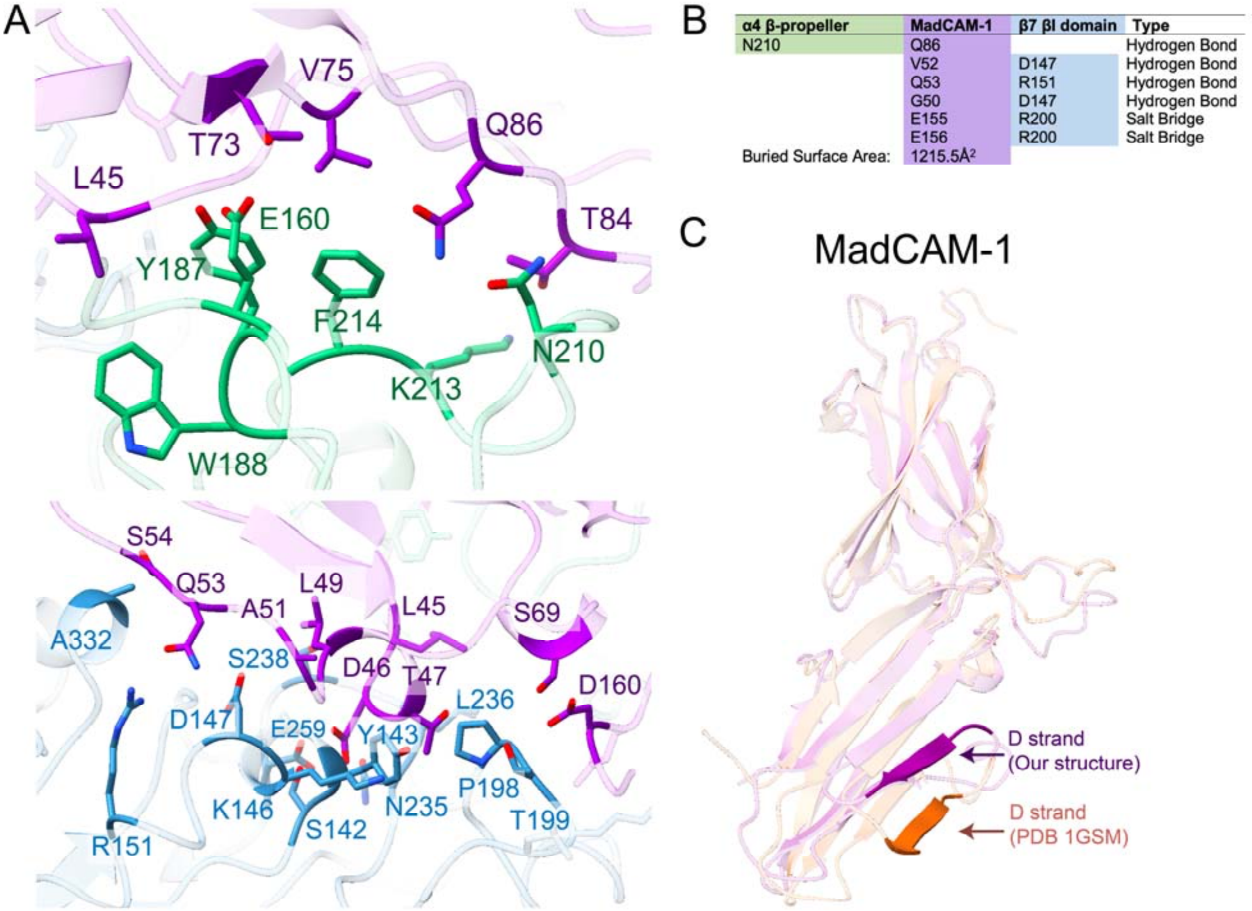

α4 β-propeller	MadCAM-1	β7 βI domain	Type
N210	Q86		Hydrogen Bond
	V52	D147	Hydrogen Bond
	Q53	R151	Hydrogen Bond
	G50	D147	Hydrogen Bond
	E155	R200	Salt Bridge
	E156	R200	Salt Bridge
Buried Surface Area:	1215.5Å²		

Supplemental Figure 3. The molecular interface between integrin α4β7 and MadCAM-1. A, MadCAM-1 (purple) binding within the α4β7 groove is stabilized by contacts with both α4 (green, top) and more extensively with β7 (blue, bottom). **B,** The PISA server[57] was used to determine electrostatic interactions in the α4β7:MadCAM-1 complex model **C,** MadCAM-1 undergoes a conformational shift upon binding to α4β7. The D strand in the first Ig-like domain of MadCAM-1 is shown in high opacity for both our structure (purple) and the crystal structure (orange, PDB 1GSM[25]). The conformation of the D strand in the crystal structure of MadCAM-1 alone would sterically clash with our integrin density.

bioRxiv preprint doi: https://doi.org/10.1101/2023.11.05.565221; this version posted November 6, 2023. The copyright holder for this preprint (which was not certified by peer review) is the author/funder, who has granted bioRxiv a license to display the preprint in perpetuity. It is made available under aCC-BY 4.0 International license.

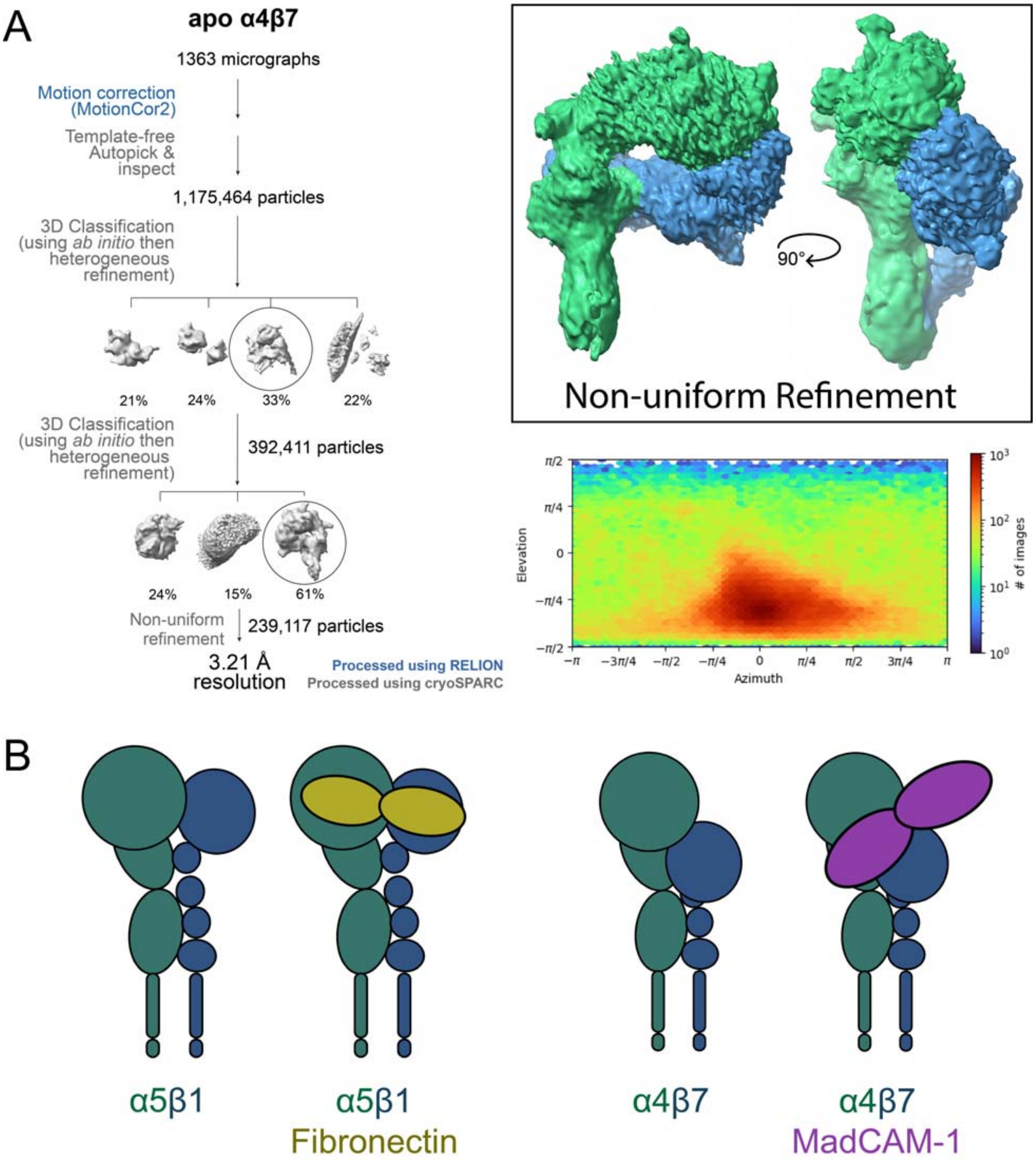

Supplemental Figure 4. Integrin α4β7 has a noncanonical activation mechanism. A, A flowchart for the data processing pipeline of apo α4β7. A preferred orientation prevents us from building an atomic model to the generated density, although secondary structure is largely preserved. **B,** Unlike integrin α5β1, the compact half-bent state of α4β7 has a significant rotation (40°) at the headpiece. This may contribute to binding to MadCAM-1, which binds α4β7 perpendicular compared to how α5β1's ligand fibronectin binds[31].

bioRxiv preprint doi: https://doi.org/10.1101/2023.11.05.565221; this version posted November 6, 2023. The copyright holder for this preprint (which was not certified by peer review) is the author/funder, who has granted bioRxiv a license to display the preprint in perpetuity. It is made available under aCC-BY 4.0 International license.

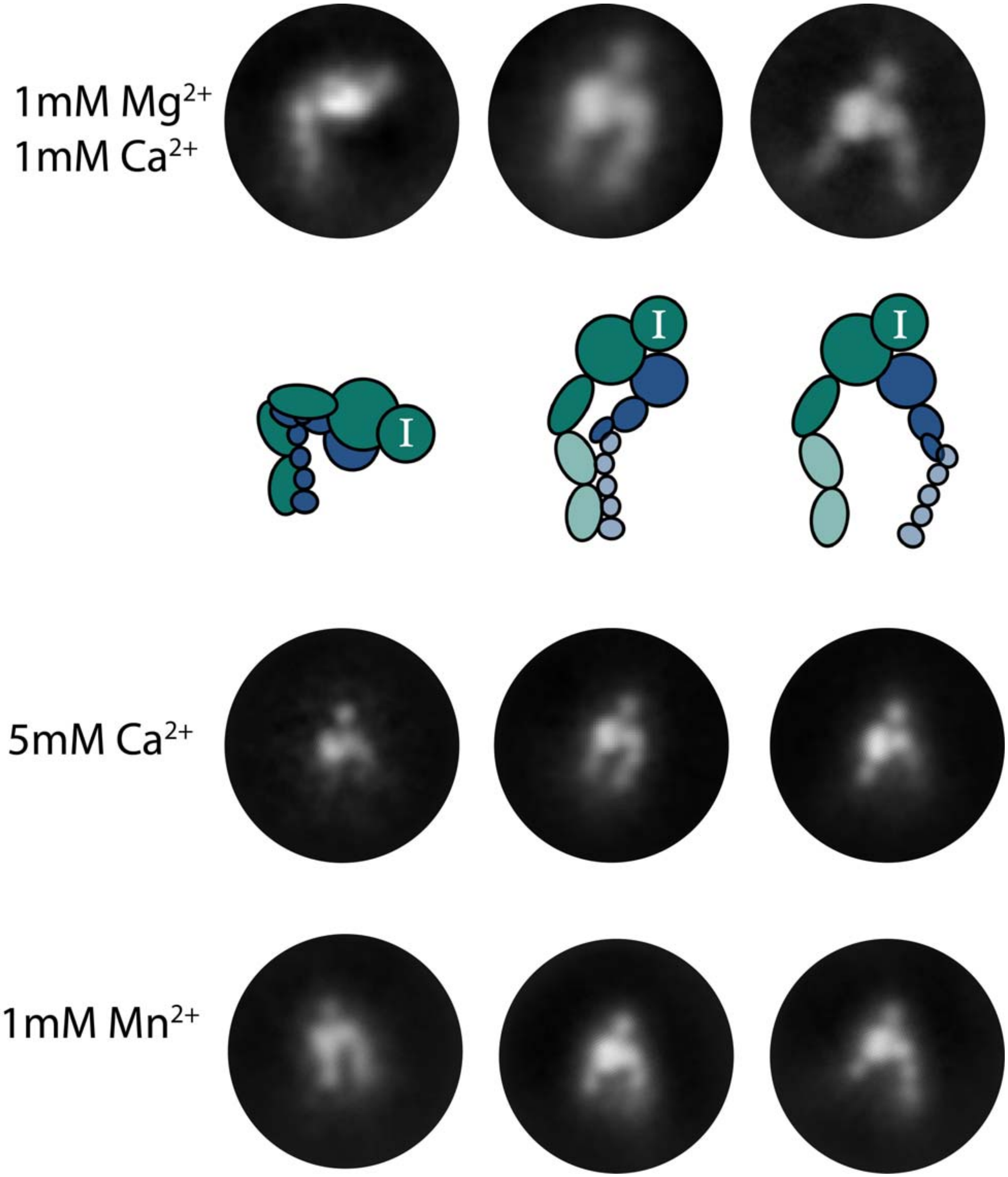

Supplemental Figure 5. Integrin αEβ7 occupies canonical conformations. Negative stain electron microscopy (nsEM) 2D class averages of integrin αEβ7 in buffers of varying ions show that αEβ7 samples conformations similar to those previously described for other integrins, although unlike other leukocyte integrins αEβ7 appears to adopt a “half-bent” conformation. The I domain is clearly resolved in each class.

bioRxiv preprint doi: https://doi.org/10.1101/2023.11.05.565221; this version posted November 6, 2023. The copyright holder for this preprint (which was not certified by peer review) is the author/funder, who has granted bioRxiv a license to display the preprint in perpetuity. It is made available under aCC-BY 4.0 International license.

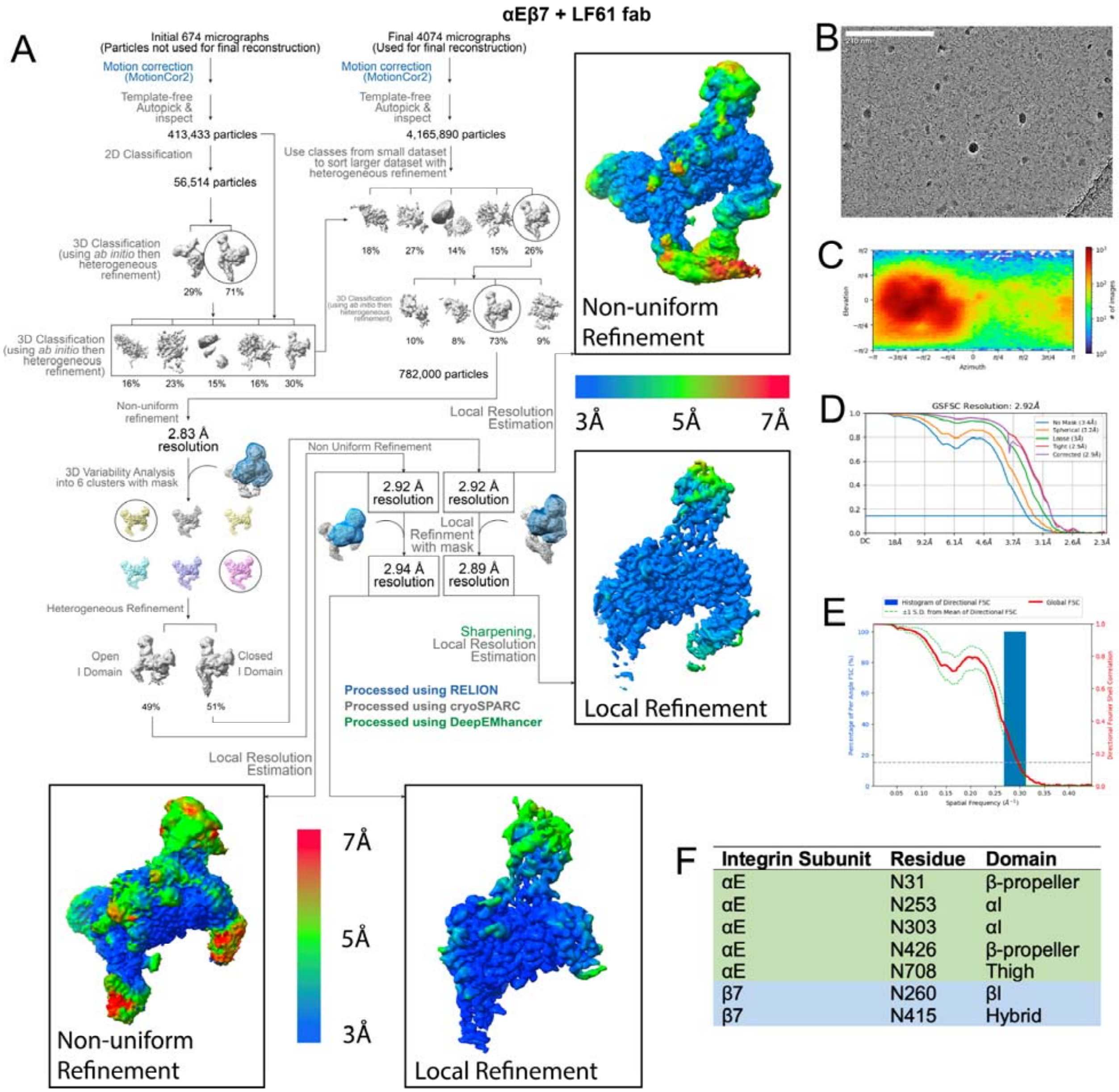

Integrin Subunit	Residue	Domain
αE	N31	β-propeller
αE	N253	αI
αE	N303	αI
αE	N426	β-propeller
αE	N708	Thigh
β7	N260	βI
β7	N415	Hybrid

Supplemental Figure 6. Data processing schematic for the αEβ7:LF61 Fab complex. A, A flowchart for the data processing pipeline of αEβ7:LF61 Fab. High-quality particles were sorted into two major classes; those that have an open I domain and those that have a closed I domain. The closed I domain structure, shown on the right with local resolution estimates, was used to model the inactive αEβ7 conformation. The open structure is shown below. **B,** Representative micrograph with 210nm scale bar, **C,** orientational distribution plot, **D,** gold-standard Fourier Shell Correlation (GSFSC) plot, and **E,** three-dimensional FSC (3DFSC) plot for the globally refined closed I domain map. **F,** Glycan residues and domains within the αEβ7:LF61 model.

bioRxiv preprint doi: https://doi.org/10.1101/2023.11.05.565221; this version posted November 6, 2023. The copyright holder for this preprint (which was not certified by peer review) is the author/funder, who has granted bioRxiv a license to display the preprint in perpetuity. It is made available under aCC-BY 4.0 International license.

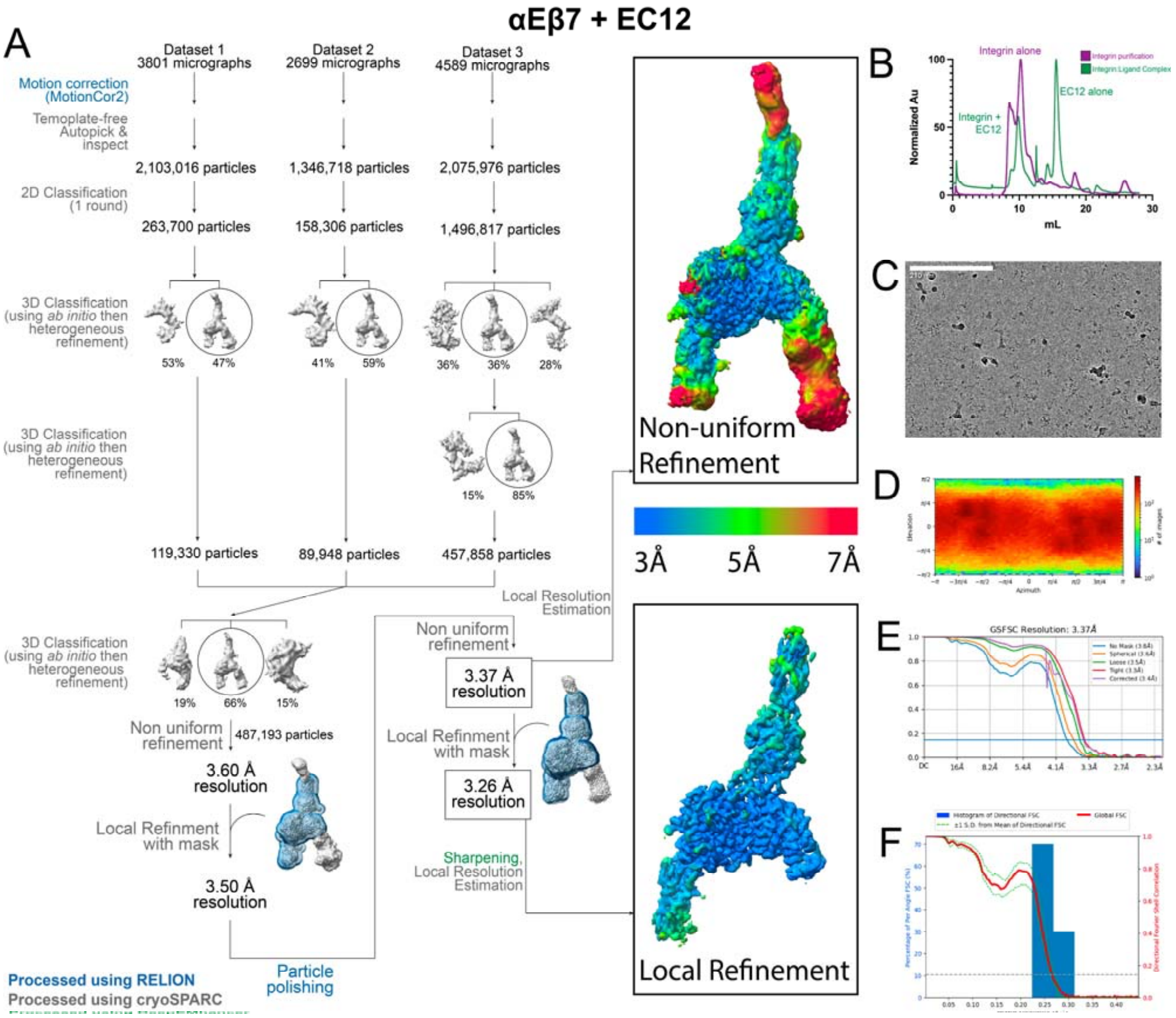

Supplemental Figure 7. Data processing schematic for the αEβ7:EC12 complex. A, Flowchart for the data processing pipeline of αEβ7:EC12. Both the global and local refinements were used for model building. **B,** Size exclusion chromatography traces showing peak shift for ligand-bound integrin. **C,** Representative micrograph with 210nm scale bar, **D,** orientational distribution plot, **E,** gold-standard Fourier Shell Correlation (GSFSC) plot, and **F,** three-dimensional FSC (3DFSC) plot the globally refined map.

bioRxiv preprint doi: https://doi.org/10.1101/2023.11.05.565221; this version posted November 6, 2023. The copyright holder for this preprint (which was not certified by peer review) is the author/funder, who has granted bioRxiv a license to display the preprint in perpetuity. It is made available under aCC-BY 4.0 International license.

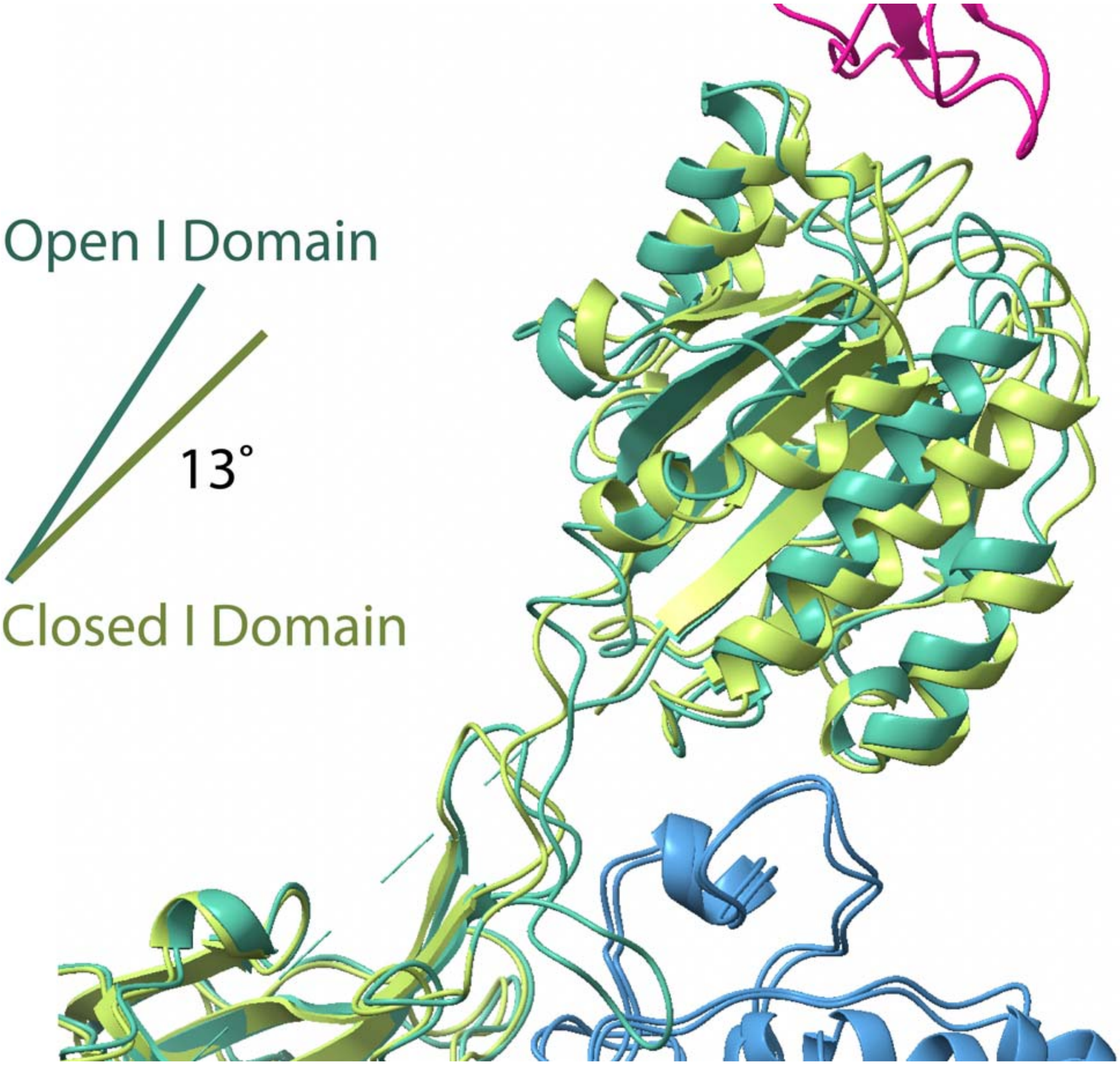

Supplemental Figure 8. Structural differences between the apo closed and ligand-bound, open αE I domain. Despite extensive structural changes the location of the I domain relative to the rest of the integrin molecule remains the same when ligand-bound. The I domain has a ~13° shift upon binding to E-cadherin. Models are aligned on the αE beta-propeller. Open I domain shown in spearmint, closed I domain shown in lime.

bioRxiv preprint doi: https://doi.org/10.1101/2023.11.05.565221; this version posted November 6, 2023. The copyright holder for this preprint (which was not certified by peer review) is the author/funder, who has granted bioRxiv a license to display the preprint in perpetuity. It is made available under aCC-BY 4.0 International license.

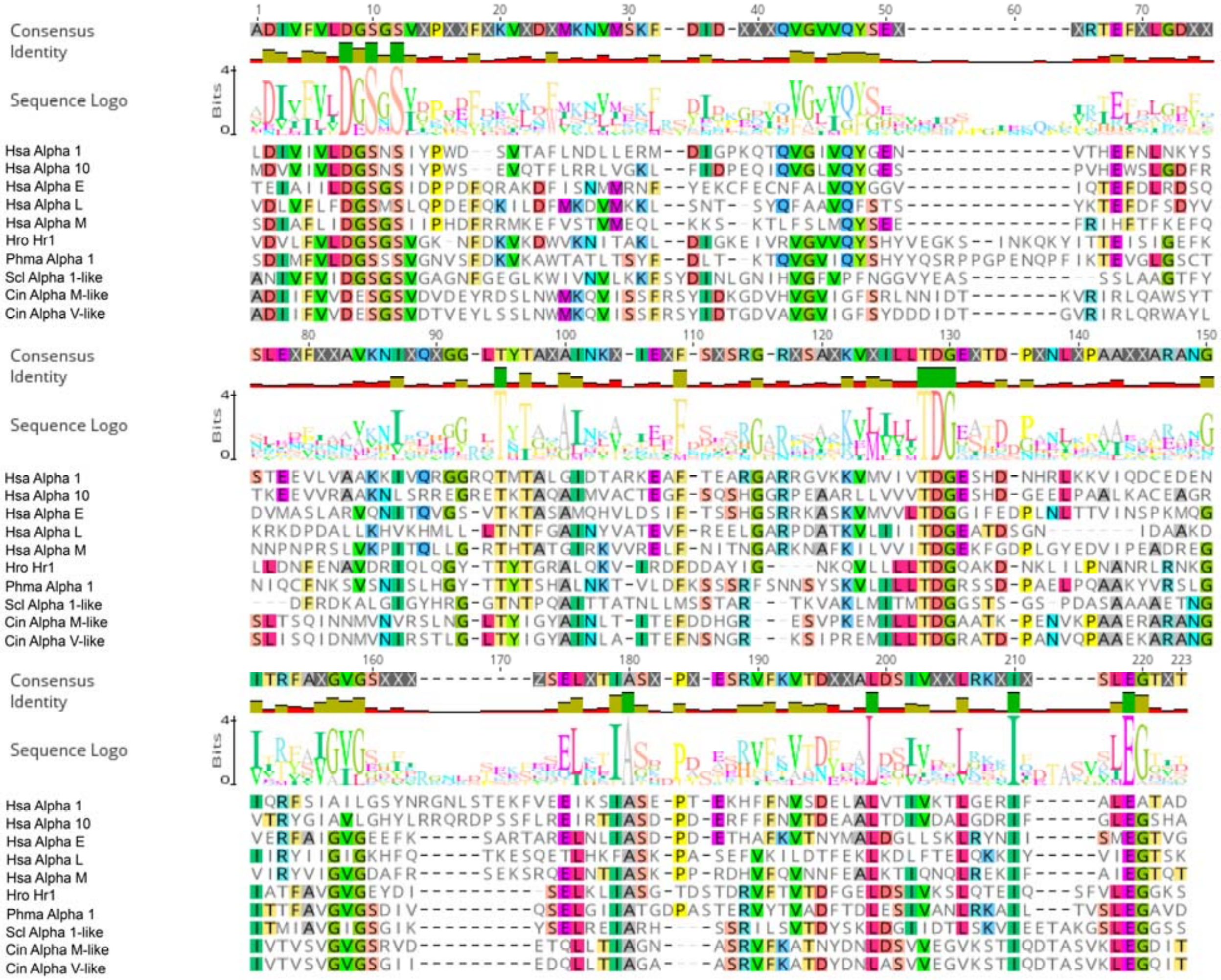

Supplemental Figure 9. An alignment of representative integrin I domains used for Hidden Markov Model (HMM)-searching. A MUSCLE alignment of integrin I domains across Olfactores was generated in Geneious. Names and accession numbers are as follows: Hsa (*Homo sapiens*) Alpha 1 (NP_852478.1), Alpha 10 (AAC31952.1), Alpha E (EAW90480.1), Alpha L (NP_002200.2), Alpha M (AAH96346.1), Hro (*Halocynthia roretzi*) Hr1 (BAB21479.1), Phma (*Phallusia mammillata*) Alpha 1 (CAB3257102.1), Scl (*Styela clava*) Alpha 1-like (XP_039274816.1), Cin (*Ciona intestinalis*) Alpha M-like (XP_026691356.1), Alpha V-like (XP_026691784.1).

bioRxiv preprint doi: https://doi.org/10.1101/2023.11.05.565221; this version posted November 6, 2023. The copyright holder for this preprint (which was not certified by peer review) is the author/funder, who has granted bioRxiv a license to display the preprint in perpetuity. It is made available under aCC-BY 4.0 International license.

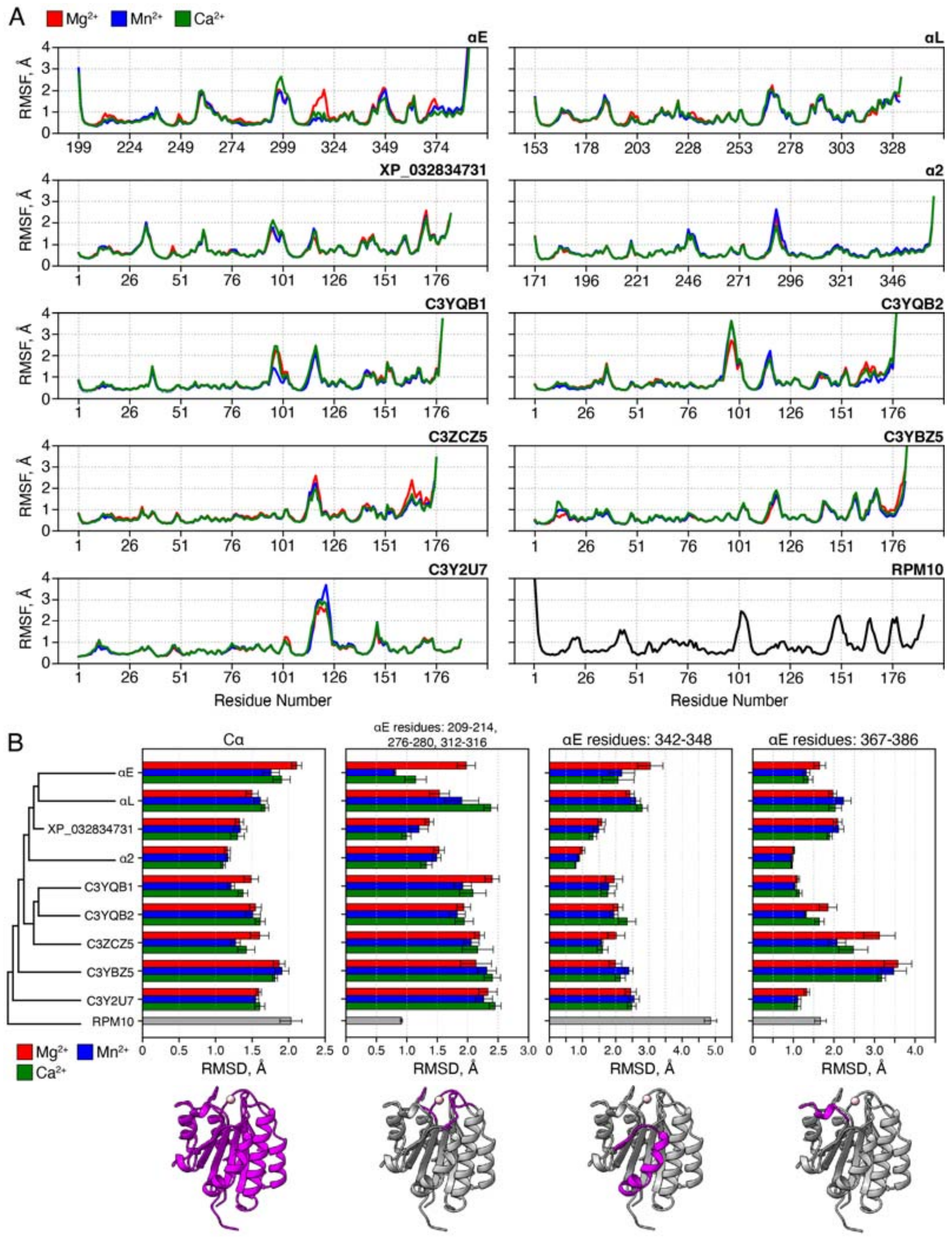

Supplemental Figure 10. Molecular dynamics simulations reveal conserved dynamics in vWFA domains. A, Root mean square fluctuation (RMSF) of 10 simulated vWFA proteins with either Mg^{2+} (red), Mn^{2+} (blue), or Ca^{2+} (green) bound in the MIDAS site. For RPN10, no ion was bound. **B,** RMSD calculation of the simulated vWFA proteins. Color represents the cation bound in the MIDAS site (Mg^{2+}, red; Mn^{2+}, blue; Ca^{2+}, green). Regions calculated, from left to right, all Cα atoms, ion coordinated loops, and sites of conformational change and colored magenta in the structure. Error bars represent standard error. For both (A) and (B), values are averaged across 10 independent replicates of 2.4μs long with only the last 2.0μs used for analysis and the initial simulation structure used as the reference.

bioRxiv preprint doi: https://doi.org/10.1101/2023.11.05.565221; this version posted November 6, 2023. The copyright holder for this preprint (which was not certified by peer review) is the author/funder, who has granted bioRxiv a license to display the preprint in perpetuity. It is made available under aCC-BY 4.0 International license.

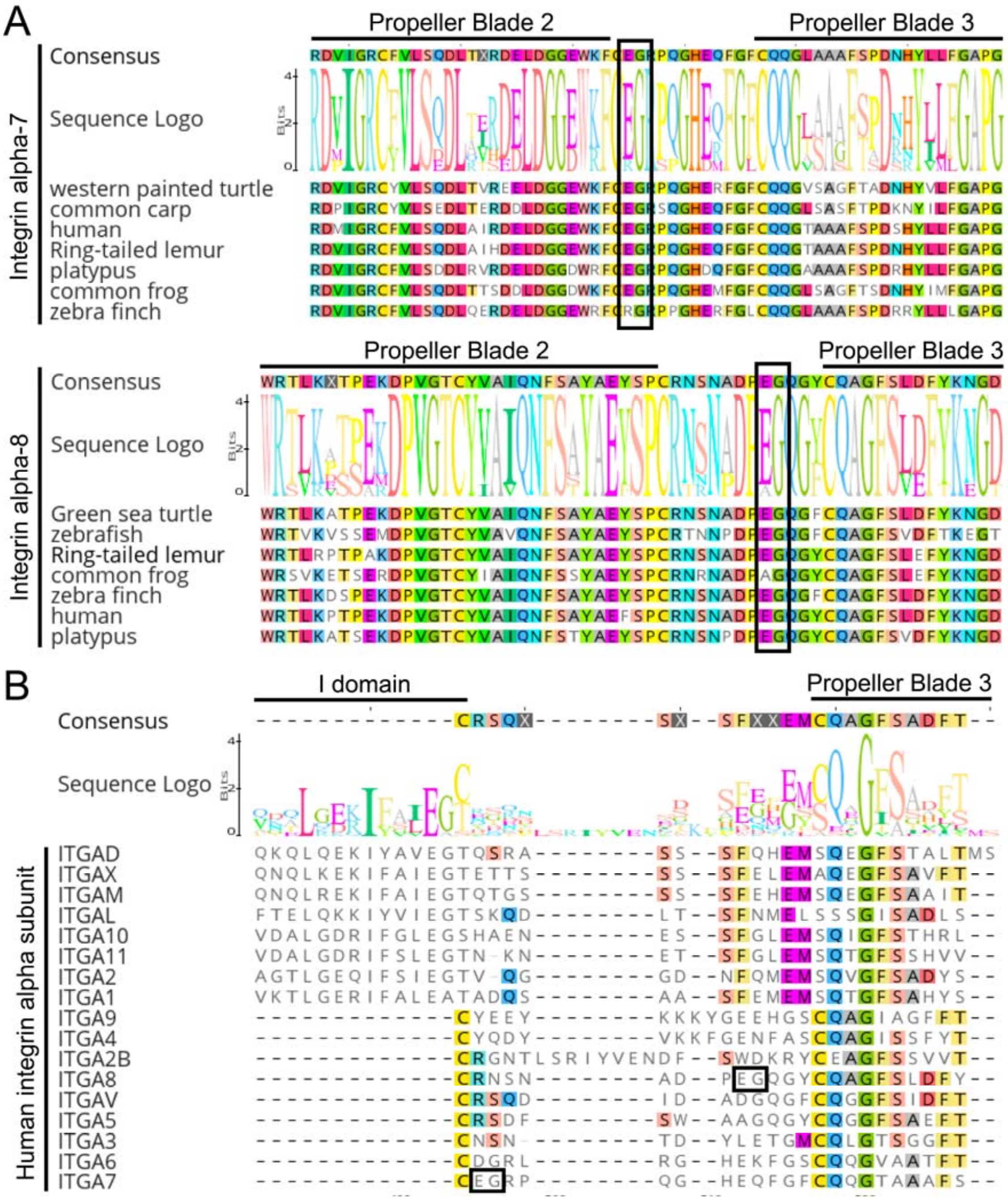

Supplemental Figure 11. Extant integrins contain internal ligand-like motifs within the beta propeller. A, The integrin I domain was inserted between the second and third beta propeller domains of an ancestral integrin. We could not find conserved “I/LEGT” motifs in our HMM search, leaving the possibility open that the I/LEGT motif was already present in the ancestral integrin. Extant integrin subunits α7 (top) and α8 (bottom) contain EG motifs that are conserved within the subunit across vertebrates. **B,** An alignment of all human integrin subunits shows these EG motifs are not broadly conserved between integrins, leaving the origin of the internal ligand I/LEGT motif ambiguous. All alignments are MUSCLE alignments generated using Geneious.

bioRxiv preprint doi: https://doi.org/10.1101/2023.11.05.565221; this version posted November 6, 2023. The copyright holder for this preprint (which was not certified by peer review) is the author/funder, who has granted bioRxiv a license to display the preprint in perpetuity. It is made available under aCC-BY 4.0 International license.

Supplemental Table 1. Collection, refinement and validation statistics for cryoEM density maps and models. Collection details for all cryoEM maps presented in the text are shown above, and validation statistics for the models subsequently generated from those maps are shown below.

	αEβ7:LF61 Closed I domain (EMDB-xxxx) (PDB xxxx)	αEβ7:LF61 Open I domain (EMDB-xxxx)	αEβ7:EC12 (EMDB-xxxx) (PDB xxxx)	α4β7:MadCAM-1 (EMDB-xxxx) (PDB xxxx)	apo α4β7 (EMDB-xxxx)
**Data collection and processing**					
Magnification	36000x	36000x	36000x	36000x	36000x
Voltage (kV)	200	200	200	200	200
Electron exposure (e–/Å^2)	50	50	50	50	50
Defocus range (μm, nominal)	1.2-1.8	1.2-1.8	1.2-1.8	1.2-1.8	1.2-1.8
Pixel size (Å)	1.122	1.122	1.122	1.122	1.122
Symmetry imposed	C1	C1	C1	C1	C1
Initial particle images (no.)	4,165,890	4,165,890	5,307,702	914,572	1,175,464
Final particle images (no.)	402,099	379,901	487,193	188,443	239,117
Map resolution (Å)	2.92	2.93	3.37	3.05	3.21
FSC threshold	0.143	0.143	0.143	0.143	0.143
Map resolution range (Å)	2.5-10.2	2.5-16.8	2.8-10.9	2.5-46.5	2.7-47.6
**Refinement**					
Initial model used (Alphafold or PDB code)	Alphafold PDB: 3V4P		Alphafold PDB: 7NLW, 4ZT1	Alphafold-multimer	
Model resolution (Å)	2.92		3.37	3.05	
FSC threshold	0.143		0.143	0.143	
Model resolution range (Å)	2.5-10.2		2.8-10.9	2.5-46.5	
Model composition					
Non-hydrogen atoms	8933		9792	9150	
Protein residues	1135		1250	1162	
Carbohydrates	22		18	22	
B factors (Å^2)					
Protein	113.5		119.7	87.1	
Ligand	N/A		N/A	N/A	
R.m.s. deviations					
Bond lengths (Å)	0.012 (11)		0.012 (12)	0.012 (10)	
Bond angles (°)	1.949 (52)		2.038 (61)	2.048 (73)	
Validation					
MolProbity score	0.78		0.82	0.97	
Clashscore	0.17		0.05	0.22	
Poor rotamers (%)	0.21		0.09	0.20	
Ramachandran plot					
Favored (%)	96.63		95.73	94.37	
Allowed (%)	3.19		3.95	5.55	
Disallowed (%)	0.18		0.32	0.09	

bioRxiv preprint doi: https://doi.org/10.1101/2023.11.05.565221; this version posted November 6, 2023. The copyright holder for this preprint (which was not certified by peer review) is the author/funder, who has granted bioRxiv a license to display the preprint in perpetuity. It is made available under aCC-BY 4.0 International license.

Supplemental Information Legends

Supplemental Video 1. Integrin α4β7 is flexible when bound to MadCAM-1. 3DFlex analysis was used to analyze continuous movement within the α4β7:MadCAM-1 complex. The β7 subunit (blue) shows hinged motion at the hybrid domain, and there is coordinated rotational movement between the α4 (green) and β7 subunits at the lower leg.

Supplemental Video 2. Compact αEβ7 stochastically samples an internally-liganded state. 3D Variability analysis was used to separate compact αEβ7:LF61 particles into states with or without the internal ligand engaged. αE is represented in green and β7 in blue. The internal ligand is the central density with high variability between frames.

Supplemental Video 3. Integrin αEβ7 is flexible when bound to E-Cadherin. 3DFlex analysis was used to analyze continuous movement within the αEβ7:EC12 complex. The active β7 subunit (blue) shows a similar hybrid domain motion as in α4β7:MadCAM-1. There is also some flexible motion between E-cadherin domains EC1 and EC2 (pink). αE is represented in green.

Supplemental Data 1. Alignment of human β-integrin protein sequences. The alignment used to generate the β-integrin subunit tree presented in Figure 2 in PHYLIP format. Names are presented as NCBI accession numbers.

Supplemental Data 2. Alignment of human α-integrin protein sequences. The alignment used to find the N- and C-terminal I domain insertion regions presented in Figures 6A and Supplemental Figure 11 in PHYLIP format. Names are presented as gene names.

Supplemental Data 3. Alignment of *Olfactores* I domain protein sequences. The alignment used to generate the Hidden Markov Model (HMM) used to identify candidate cephalochordate I domain-like proteins in FASTA format. Names for human genes are given "Alpha*," while all other names are presented as NCBI accession numbers.

Supplemental Data 4. Alignment of I domains and cephalochordate outgroup vWFA domains. The alignment used to generate the phylogenetic tree presented in Figure 6C. Names are presented as NCBI accession numbers for integrin I domains or Uniprot identifiers for cephalochordate vWFA domains.
